# Semantic Web for data harmonization in Chinese medicine

**DOI:** 10.1186/1749-8546-5-2

**Published:** 2010-01-12

**Authors:** Kei-Hoi Cheung, Huajun Chen

**Affiliations:** 1 Yale Center for Medical Informatics and Departments of Anesthesiology and Genetics, School of Medicine, Computer Science Department, Yale University, New Haven, CT 06510, USA; 2 College of Computer Science, Zhejiang University, Hangzhou, Zhejiang, 310027, PR China

## Abstract

Scientific studies to investigate Chinese medicine with Western medicine have been generating a large amount of data to be shared preferably under a global data standard. This article provides an overview of Semantic Web and identifies some representative Semantic Web applications in Chinese medicine. Semantic Web is proposed as a standard for representing Chinese medicine data and facilitating their integration with Western medicine data.

## Background

As the scientific evidence for the preventive and therapeutic efficacy of Chinese medicine (CM) is growing, it is strongly demanded to bridge CM with Western medicine (WM), particularly through the data obtained from biomedical and clinical research. For example, there were acupuncture studies on certain diseases/disorders such as chronic pain [1,2] and cerebral palsy [3], on pharmacological, molecular and therapeutic properties of various Chinese herbs [4,5] using high-throughput technologies such as DNA microarray and mass spectrometry [6,7]. Technical challenges include not only the increasing amount of CM literature but also the wide variety of data among various databases. Some representative databases are as follows:

i) TCMGeneDIT [8] is a database containing disease-gene-herb associations as the results of mining the biomedical literature;

ii) Phytochemical databases of Chinese herbal constituents were constructed [9];

iii) ClinicalTrials http://clinicaltrials.gov/ contains information of a large collection of clinical trials including those that involve CM;

iv) MedlinePlus http://www.nlm.nih.gov/medlineplus/[10] developed by the United States National Library of Medicine provides consumers and health professionals with research information which covers certain herbal supplements;

v) TCM Online http://cowork.cintcm.com/engine/windex1.jsp consists of over 40 categories of CM Databases such as the Traditional Chinese Medical Literature Analysis and Retrieval Database, Clinical Medicine Database, Traditional Chinese Drug Database, Database of Chinese Medical Formula, Traditional Chinese Medicine Enterprises and Productions Database, and State Standards Database.

Data mining and integration of CM and WM databases are of great value but problematic [9,11]. Data mining and integration problems include heterogeneity in data formats and structures as well as a lack of standard terminology. Cultural and linguistic differences further complicate data integration. In the informatics community, methods developed for data integration can be categorized into: (1) data warehousing to translate data and (2) query federation to translate query. Both approaches have their pros and cons. For example, the data warehousing approach has good query performance as data are queried locally, but data are not always up-to-date (data updates are to be made periodically to keep the warehouse in synchrony with the member data sources). The query federation approach guarantees data to be up-to-date, but it may suffer from query performance especially when large volumes of data are queried and joined over the network. Despite their differences, these approaches are based on a common data model. The use of such a model is feasible in either a single enterprise or a small group of enterprises. A common data model which can overcome national, geographical, and cultural boundaries would be different without a global data representation standard. To this end, Semantic Web [12] has the potential to help realize data harmonization in CM.

## Semantic Web and its applications in Chinese medicine

Semantic Web is an evolving extension of the World Wide Web in which the semantics of information and services on the Web are defined, making it possible for the Web to "understand" and answer queries in accordance with the Web content. SW's enabling technologies include the Uniform Resource Identifier (URI) http://www.w3.org/Addressing/ and Resource Description Framework (RDF) http://www.w3.org/RDF/, which are the Semantic Web standards for data identification and data representation respectively. The RDF provides a "triple" format for representing a statement that consists of a subject, property and object. Each component of the triple is identified by a URI that serves as a global unique identifier for the Web. For example, the following triple (statement) asserts that an herb-derived drug "Huperzine A" (subject) "inhibit" (property) "NMDA receptor" (object).

**Subject -- **http://en.wikipedia.org/wiki/Huperzine_A

**Property -- **http://en.wikipedia.org/wiki/inhibit

**Object -- **http://en.wikipedia.org/wiki/NMDA_Receptor

The above example demonstrates that the Wikipedia URIs are used to identify and define the subject, property and object (this is only for demonstration purposes). The statement indicates an "inhibitory" effect of the drug "Huperzine A" on "NMDA Receptor" (drug target). A collection of linked RDF statements forms a directed acyclic graph (DAG). Such collections of statements represent the knowledge of a domain. To query and manipulate RDF statements, we may use "SPARQL" http://www.w3.org/TR/rdf-sparql-query/, which is the RDF query language standard. SPARQL is analogous to SQL http://en.wikipedia.org/wiki/SQL for querying relational databases.

To capture richer data semantics to support computational inference and reasoning, the RDF Schema (RDFS) http://www.w3.org/TR/rdf-schema/ and the Web Ontology Language (OWL) http://www.w3.org/TR/owl-features/ have been used to encode ontologies in the biomedical domains [13,14]. RDFS provides the *rdfs:Class *construct to declare a resource as a class, e.g. Herb. A hierarchy of classes can be defined using the *rdfs:subClassOf *construct. For example, "Huperzia serrata" is a subclass of "Herb". Most of the RDFS components are included OWL, which is more expressive than RDFS. OWL has the built-in property *owl:sameA*s that allows a synonymous relationship between two classes (e.g. "Huperzine A" and "Huperzia serrata"). Cardinality constraints can be applied to properties (e.g. the "inhibit" property can have a minimum cardinality of one and cardinality with a maximum of a positive integer). While OWL is semantically richer than RDF or RDFS, it can be expressed using the RDF syntax. OWL reasoners such as Pellet [15] and Racer [16] can be used to make inferences out of OWL ontologies.

Adoption of the Semantic Web has been significantly important to health care and life sciences. In part, the adoption has been driven by the World Wide Web Consortium (W3C), which launched the Semantic Web for Health Care and Life Sciences Interest Group (HCLS IG) http://www.w3.org/2001/sw/hcls/. The group has been chartered to develop, adopt, and support the use of Semantic Web technologies and practices to improve collaboration, research and development in health care and the life sciences.

As RDF/OWL-formatted datasets are growing in terms of the number and size , efficient data storage and manipulation become big issues. To this end, a variety of triplestore technologies have emerged, including Virtuoso http://virtuoso.openlinksw.com/, Oracle http://www.oracle.com/technology/tech/semantic_technologies, AllegroGraph http://agraph.franz.com/allegrograph/, and Sesame http://www.openrdf.org/. While some of these technologies (e.g. Oracle and Virtuoso) are proprietary, others (e.g. Sesame) are open source. Some of them (e.g. Virtuoso, AllegroGraph and Sesame) support SPARQL, but some others (e.g. Oracle) have their own RDF query languages. To provide a uniform query access, many triplestores provide a so-called "SPARQL endpoint" so that queries can be issued by client programs against the triplestores via the SPARQL language. For example, even though Oracle does not support SPARQL internally, it can be configured to provide an external SPARQL endpoint through the Jena adaptor http://www.oracle.com/technology/tech/semantic_technologies/htdocs/documentation.html. Triplestores such as Oracle provide their own native OWL reasoners, while some others (e.g., Sesame) can be integrated with external reasoners.

Linked Data [17] is a new method of exposing, sharing, and connecting data via dereferenceable HTTP URI's on the Semantic Web. A dereferenceable HTTP URI serves as both an identifier and a locator. The key idea is that useful information should be provided to data consumers when its URI is dereferenced. Using the Linked Data approach, not only do data providers make their data available in the form of RDF graphs, but data linkers can also create new RDF graphs that consist of links between independently developed RDF graphs provided by different sources. Examples of Linked Data, e.g. DBpedia http://wiki.dbpedia.org/OnlineAccess, are listed on Linking Open Data (LOD) http://esw.w3.org/topic/SweoIG/TaskForces/CommunityProjects/LinkingOpenData. A similar effort has been launched by the Linking Open Drug Data task force of the HCLS IG to use the linked data approach to link drug-related data.

As the relational database technology is prevalent in the health care and life science domains, many of the CM databases are currently in the relational format. While these relational databases serve the specific needs of individual labs or institutions, their accessibility by other labs or institutions is limited. An object or data record is identified by a unique identifier (primary key) that is local to the database. In other words, the same identifier does not identify the same object (data records) in different relational databases. Another issue with the relational databases is that relationships are defined based on links between primary and foreign keys. These links are not to convey some meaning semantically. Semantic Web can be used to address this problem by allowing a semantic layer to be created on top of existing relational databases. Semantically rich queries (based on meaningful relationship names) can be formulated at the semantic layer (built using the Semantic Web technology) and then be mapped to the local queries against the underlying relational databases. DartGrid [18] is a system demonstrating the use of this semantic web approach to integrate CM databases. The advantage of this approach is that existing relational databases and applications accessing these databases need not be abandoned, while new powerful applications can be developed to make use of the Semantic Web features.

As data are increasingly available in RDF/OWL format, new warehouses and federated query systems have been built from scratch using Semantic Web technologies to allow direct access by programs. As part of the HCLS IG effort, a subset of TCMGeneDIT was converted into RDF format and loaded into an RDF triplestore [19]. In addition, the BioRDF task force of the HCLS IG has undertaken the effort of implementing query federation using the Semantic Web [20].

Ontologies encoded by Semantic Web enable expressive knowledge representation, integration, and discovery. Ontology research is active in the biomedical informatics community. Examples include the OBO Foundry [21] and BioPortal [22] that provide access to a large collection of biomedical ontologies. These ontologies are relevant to CM research especially when relating CM to WM. In addition, efforts have begun to create new ontologies specifically for CM. For example, China Academy of Traditional Chinese Medicine has created a CM ontology that defines more than 8,000 classes and over 50,000 instances and may help integrate heterogeneous and disparate databases [23].

Some information technologies such as text mining, Grid computing, and Web services have been using the Semantic Web. These technologies combined with the Semantic Web can further empower CM researchers to carry out *in silico *research.

## Discussion

Given the long history of CM, most of the CM documents were written in Chinese. While the Web is multilingual, a simple literal translation, however, is not sufficient in terms of making the CM knowledge accessible by Western researchers. An example is the translation of signs and symptoms between CM and WM. For example, the term *Re *(which literally means "Heat") in CM may be referred to as high fever and irritability in WM. The theories behind WM and various CM can be fundamentally different, leading to the difficulty to make alignments among their domain ontologies. For example, CM practitioners interpret human body and organs based on Chinese philosophical ideas of "yin-yang" and "five-elements". They are aware of the efficacy of the herb, Huperzia serrata (HS), in aging disorders, and interpret the action mechanism of this herb as strengthening the *Shen *(kidney). Biomedical scientists analyze some experimental evidence, and deduce that a compound of the herb HS acting on the brain can serve as a potential therapy for the Alzheimer's disease. In this case, HS targets the brain (WM) instead of the *Shen *(kidney).

These language gaps limit the communication and interaction between WM and CM in both directions. On the one hand, scientific communities have not reached the full potential of utilizing CM knowledge. On the other hand, best practices of WM are not widely adopted in the regions where CM is predominant form of healthcare service. To bridge these gaps, we need to establish an infrastructure that can support communication and collaboration in integrative medicine studies. The infrastructure should also be able to capture and publish the results of these integrative medicine studies to extend the actionable knowledge shared among communities.

Data sharing is a key to advancing science in the digital age [24]. For example, the Human Genome Project [25] made public release of data to the scientific community. This open access culture should be widely encouraged and supported by the CM community. At the same time, we need to address the concerns of sharing data. Among these concerns is the intellectual property including data ownership, attribution, and licensing. The legal complication should never be underestimated, as the laws affecting data sharing vary from one country to another. The Consortium for Globalization of Chinese Medicine http://www.tcmedicine.org/ was formed to promote data sharing as well as collaboration among academia, industry and regulatory agencies in various countries.

While the Semantic Web is a candidate for standardizing the format of CM data sharing, it needs to be used in conjunction with other standardization efforts that are underway in the CM community, e.g. the regulatory standards for quality control of Chinese medicinal materials [26]. This also brings up the question of how much information needs to be provided for describing different types of CM data for reproducibility, quality, and safety purposes. In the fields of genomics and proteomics, standards such as MIAME [27] and MIAPE [28] are available for specifying the minimum amount of information to be provided for microarray experiments and proteomics experiments, respectively. Similar standards are needed for sharing scientific data in CM.

There is a broad spectrum of international Semantic Web research related to the health care and life sciences. Semantic Web research effects in CM are mainly in Asia. It would be beneficial to integrate CM into these international activities. More use cases are needed to demonstrate how the Semantic Web can be used to harmonize CM and WM through data linking and integration as well as community collaboration.

## Concluding remarks

As the interest of using Semantic Web in the health care and life sciences is growing, it has the potential to facilitate cross-disciplinary data integration between Chinese Medicine and Western Medicine. The Semantic Web could potentially play an important role in Chinese medicine informatics involving a new breed of informaticians who are able to bridge multiple scientific and cultural disciplines.

## Competing interests

The authors declare that they have no competing interests.

## Authors' contributions

Both authors took part in the discussion and writing of this article. They also read and approved the final version of the manuscript.
